# P-337. HIV drug resistance-associated mutations within molecular clusters of rapid HIV transmission, 2018–2023

**DOI:** 10.1093/ofid/ofaf695.556

**Published:** 2026-01-11

**Authors:** Katherine C McNabb, Neeraja Saduvala, Kathryn Curran, Richard Teran, Anne Marie France, Walid Heneine, Rachael Billock, Joel Wertheim, Angela L Hernandez, Alexandra Oster

**Affiliations:** Centers for Disease Control and Prevention, At, Georgia; SeKON, Atlanta, Georgia; Centers for Disease Control and Prevention, At, Georgia; Centers for Disease Control and Prevention, At, Georgia; Centers for Disease Control and Prevention, At, Georgia; Centers for Disease Control and Prevention, At, Georgia; Centers for Disease Control and Prevention, At, Georgia; University of California, San Diego, San Diego, California; Centers for Disease Control and Prevention, At, Georgia; Centers for Disease Control and Prevention, At, Georgia

## Abstract

**Background:**

CDC identifies and responds to clusters of rapid HIV transmission using HIV molecular data reported to the National HIV Surveillance System. Immediate initiation of antiretroviral therapy (ART) and pre-exposure prophylaxis (PrEP) for partners is essential in response efforts, but drug resistance-associated mutations (DRAMs) can undermine effectiveness. This study characterized DRAMs impacting first-line ART and PrEP regimens among HIV sequences from individuals within molecular clusters to inform response initiatives.

**Figure:**
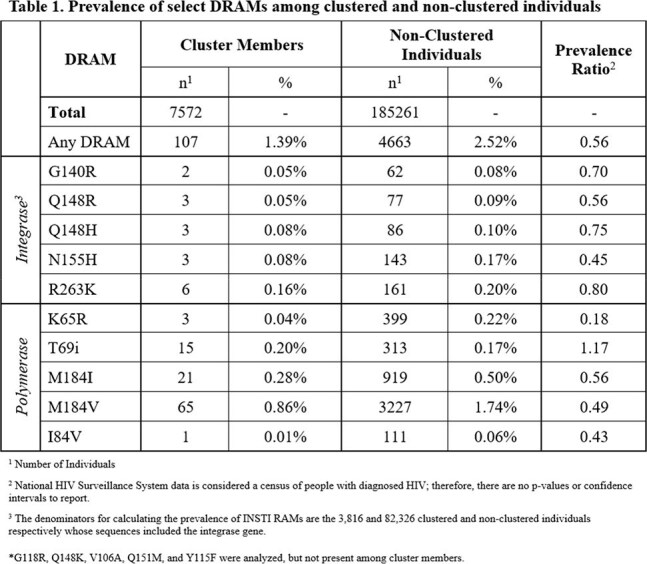

**Methods:**

Using HIV molecular sequence data from individuals diagnosed with HIV from 2015 to 2023, we assessed the prevalence of select DRAMs among members of molecular clusters identified from 2018 to 2023, as well as among non-clustered individuals. Further, we investigated clusters for extensive DRAM prevalence, defined as ≥ 50% of cluster members with a specific DRAM. Clusters with rapid HIV transmission were defined as those with ≥ 5 diagnoses in the past 12 months (≥ 3 diagnoses in low morbidity jurisdictions) using a genetic distance of ≤ 0.005 substitutions per site among people with HIV diagnosed during the prior three years. This analysis focused on DRAMs in the integrase and polymerase genes conferring intermediate- or high-level resistance to bictegravir, cabotegravir, dolutegravir, darunavir, tenofovir, emtricitabine, or lamivudine.

**Results:**

Our analysis included 192,833 persons with diagnosed HIV, including 7,572 people in 404 molecular clusters and 185,261 non-clustered individuals. The overall prevalence of individuals with at least one DRAM was 1.39% (n=107) and 2.52% (n=4,663) among clustered and non-clustered individuals, respectively (Table 1). No clusters showed extensive DRAM prevalence, and the highest prevalence of a DRAM within any cluster was 20%.

**Conclusion:**

The prevalence of DRAMs affecting first-line ART and PrEP regimens among cluster members was low, and cluster members had lower prevalence of DRAMs than non-clustered individuals. No clusters exhibited extensive DRAM transmission, indicating rapid transmission of DRAMs in molecular clusters does not currently pose a public health threat. However, ongoing monitoring of clusters for extensive DRAM transmission may still be prudent to inform response strategies.

**Disclosures:**

All Authors: No reported disclosures

